# Development and External Validation of a Dynamic Nomogram With Potential for Risk Assessment of Ruptured Multiple Intracranial Aneurysms

**DOI:** 10.3389/fneur.2022.797709

**Published:** 2022-02-08

**Authors:** TingTing Chen, WeiGen Xiong, ZhiHong Zhao, YaJie Shan, XueMei Li, LeHeng Guo, Lan Xiang, Dong Chu, HongWei Fan, YingBin Li, JianJun Zou

**Affiliations:** ^1^School of Basic Medicine and Clinical Pharmacy, China Pharmaceutical University, Nanjing, China; ^2^Department of Clinical Pharmacology, Nanjing First Hospital, Nanjing Medical University, Nanjing, China; ^3^Department of Neurology, The First Affiliated Hospital (People's Hospital of Hunan Province), Hunan Normal University, Changsha, China; ^4^Department of Neurology, The First Affiliated Hospital, Hunan University of Medicine, Huaihua, China; ^5^Department of Neurosurgery, The Second Affiliated Hospital, Nanjing Medical University, Nanjing, China; ^6^Department of Pharmacy, Nanjing First Hospital, China Pharmaceutical University, Nanjing, China

**Keywords:** multiple intracranial aneurysms, dynamic nomogram, web-based calculator, rupture, risk assessment

## Abstract

**Background and Purpose:**

About 20.1% of intracranial aneurysms (IAs) carriers are multiple intracranial aneurysms (MIAs) patients with higher rupture risk and worse prognosis. A prediction model may bring some potential benefits. This study attempted to develop and externally validate a dynamic nomogram to assess the rupture risk of each IA among patients with MIA.

**Method:**

We retrospectively analyzed the data of 262 patients with 611 IAs admitted to the Hunan Provincial People's Hospital between November 2015 and November 2021. Multivariable logistic regression (MLR) was applied to select the risk factors and derive a nomogram model for the assessment of IA rupture risk in MIA patients. To externally validate the nomogram, data of 35 patients with 78 IAs were collected from another independent center between December 2009 and May 2021. The performance of the nomogram was assessed in terms of discrimination, calibration, and clinical utility.

**Result:**

Size, location, irregular shape, diabetes history, and neck width were independently associated with IA rupture. The nomogram showed a good discriminative ability for ruptured and unruptured IAs in the derivation cohort (AUC = 0.81; 95% CI, 0.774–0.847) and was successfully generalized in the external validation cohort (AUC = 0.744; 95% CI, 0.627–0.862). The nomogram was calibrated well, and the decision curve analysis showed that it would generate more net benefit in identifying IA rupture than the “treat all” or “treat none” strategies at the threshold probabilities ranging from 10 to 60% both in the derivation and external validation set. The web-based dynamic nomogram calculator was accessible on https://wfs666.shinyapps.io/onlinecalculator/.

**Conclusion:**

External validation has shown that the model was the potential to assist clinical identification of dangerous aneurysms after longitudinal data evaluation. Size, neck width, and location are the primary risk factors for ruptured IAs.

## Introduction

The incidence of intracranial aneurysms (IAs) is approximately 3%, which can result in aneurysmal subarachnoid hemorrhage with a high mortality and disability rate (1, 2). Multiple intracranial aneurysms (MIAs), defined as coexisting ≥2 IAs, occur in about 20.1% of IAs carriers (3). Previous studies have shown that MIAs are more prone to grow and rupture (4–6). Among patients over 70 years old, a worse prognosis occurs in those with MIAs than those with a single IA (7). However, for patients with unruptured MIAs, the strategy of all treatment or no treatment may be expensive or ineffective. Therefore, physicians usually need to consider the rupture risk of each IA when formulating treatment strategies for patients with MIA. It is of great clinical significance to identify the IAs with a high risk of rupture among patients with MIA.

As one of the widely accepted scoring systems, the PHASES had been developed to estimate the rupture risk of IAs (8). Despite that, most of the cases in this study were single IA patients. Some possible differences exist between single IA and MIAs patients, such as the potential additive effect (9). It may be related to the relative risk of each IA and the number of increased coexisting IAs. Besides, it only took the largest IA in MIAs patients into account while ignoring other coexisting IAs. Considering that the responsible IA in one-third of MIA patients is not the largest (10), this score may not be well-suitable for patients with MIA.

In addition, several prediction models (11, 12) including hemodynamic parameters and radiomics features were also established to evaluate the rupture risk of IAs and seemed to bring some facilitation to physicians. But, the acquisition of these data usually required some particular software and complex measurement methods, which may bring much pressure to the already busy daily work of physicians and limit the practicability of models in a clinical environment.

The purpose of this study was to find connections between readily accessible features and ruptured IAs. We also attempt to develop and externally validate a feasible dynamic nomogram model to assess the rupture risk of each IA among MIAs patients, and then provide a reference for clinicians when they are faced with developing treatment strategies for patients with unruptured aneurysms at admission.

## Method

### Study Population

After obtaining the permission from the institutional ethics committee, we retrospectively analyzed the data of all consecutive patients with IA admitted to the Hunan Provincial People's Hospital between 2015 and 2021 who met the criteria as follow: (1) no less than two aneurysms; (2) complete neuroradiological examination by DSA imaging; and (3) confirmed ruptured aneurysm by intraoperative findings or computed tomography scan imaging. Patients were excluded if they had fusiform or dissecting IAs, uncertain rupture location, and incomplete clinical and imaging data. To construct an external validation cohort, we also collected data from the Second Affiliated Hospital of Nanjing Medical University between 2009 and 2021 in accordance with the above inclusion and exclusion criteria.

### Data Collection

The following clinical data were collected from medical records: age; sex; history of smoking, drinking, hypertension, diabetes mellitus (DM), hyperlipidemia (HLP), atrial fibrillation, coronary heart disease (CHD), and subarachnoid hemorrhage (SAH).

Morphological parameters of IAs were extracted from three-dimensional (3D) DSA images. Data was collected by one senior neurosurgeon and was supervised by two others, and the disagreement was discussed to reach a consensus. The aneurysmal size was defined as the maximum distance within the aneurysm sac. Size and neck width was measured on a scale of 0.1 mm. Sidewall location or bifurcation location were categorized according to the relative position of the IA to the parent vessel. The presence of aneurysm wall protrusions, bi- or multi-lobular, or small blebs seemed like irregular shapes. Aneurysmal location was recorded as 5 categories: anterior cerebral artery (ACA, including anterior communicating artery), internal carotid artery (ICA), middle cerebral artery (MCA), posterior communicating artery (PCOA), and posterior circulation (PC, including posterior cerebral artery [PCA], vertebral artery [VA], posterior inferior cerebellar artery [PICA], basilar artery [BA], and other posterior circulation location).

### Statistical Analysis

Statistical analyses were conducted using SPSS version 25 (IBM Corporation, USA), STATA version 13 (Stata Corporation, College Station, TX, USA), and R version 4.1 (http://www.R-project.org/). Continuous and categorical variables were described as mean ± *SD* and percentage, respectively. Comparison of the differences between two groups was performed using Mann-Whitney U test or Student's *t*-test for continuous data, and Chi-squared test for categorical data. A two-sided *P* < 0.05 was a statistically significant level.

A mixed-effects logistic regression model was considered because each patient harbored at least two IAs and the outcome was binary. We firstly developed a null model with patient ID as a random effect and calculated the intra-class correlation coefficient (ICC) of this model (13–15). The ICC measures the degree of clustering in our data by reporting the ratio of intergroup variance to the total variance. If ICC is significantly >0, a multi-level analysis is needed.

Univariate and multivariate analyses of the derivation cohort were carried out for the identification of risk factors. Firstly, the associations within the risk of IA rupture among patients with MIAs and with clinical and morphological characteristics were tested by univariable analysis. Then the variables with *P* < 0.1 or thought to be independent risk factors of IA rupture in clinical practice were analyzed by multivariable logistic regression (maximum likelihood method). After a forward stepwise selection procedure (elimination criterion: *P* > 0.1; enter criterion: *P* < 0.05), the variables that were retained and entered into the final regression were used to model the probability of IA rupture in patients with MIAs. Eventually, the associations of risk factors with rupture status were reported as odds ratios (ORs) with 95% CIs, and the nomogram model was graphically visualized as a nomogram through and then was developed into an online calculator called a dynamic nomogram through “Dynnom” package.

The performance of the nomogram was assessed in the derivation and external validation set in respect of discrimination, calibration, and clinical utility. The discrimination ability was quantitatively assessed by calculating the C statistic. Calibration measures how closely the predicted probabilities agree numerically with the actual outcomes based on Hosmer–Lemeshow goodness-of-fit test and a calibration plot. Decision curve analysis was used to explore the net benefit (NB) of the nomogram model, and the true-positive rate (TPR) and false-positive rate (FPR) classifications were considered at increasing decision thresholds, where the NB = TPR - FPR ^*^ threshold probability/ (1-threshold probability).

## Result

### Patient Characteristics

This retrospectively multicentric study totally included 262 patients with 611 IAs in the derivation cohort and 35 patients with 78 IAs in the external validation cohort. Two patient selection flow charts see Supplementary Figures S1, S2. No significant differences were noted between the derivation and external validation cohort (*P* > 0.05), except for age (*P* = 0.017), diabetes history (*P* = 0.015) and shape (*P* = 0.002), and number of IAs (P = 0.03). Besides, the proportion of ruptured IA was 32.2% (197/611) and 28.8% (21/78) in the derivation and external validation cohort, respectively. In the derivation cohort, 17.3% (34/197) of ruptured IAs were the smaller IA rather than the largest IA in the coexisting aneurysms. Besides, 75.6% (149/197) of ruptured IAs were small IA with size ≤ 7 mm. The clinical and morphological characteristics of the derivation and external validation cohort were summarized in Supplementary Table S1.

### Selected Risk Factors for Model

Patient-level variability was assessed by ICC. As shown in Supplementary Table S2, the ICC value in the null model was 2.416 × 10^−15^, which indicates that there was no clustering or community level variability of IA rupture. Hence, we employed conventional logistic regression rather than mixed-effects logistic regression to develop a model to assess the IA rupture risk among patients with MIA.

In the derivation cohort, the univariable analysis showed the significant differences in diabetes history (*P* = 0.031), shape (*P* < 0.001), number of IAs (*P* = 0.021), size (*P* < 0.001), and location (*P* < 0.001) between the unruptured and ruptured group (Table 1). Subsequently, the six variables with *P* < 0.1 on univariable analyses and four independent risk factors in previous studies (age, hypertension history, SAH history, and bifurcation location) were analyzed by the multivariable logistic regression analyses. After a process of forward stepwise model selection, as present in Table 2, the following five independent predictors of MIA rupture were identified: irregular shape (OR: 3.011, 95% CI: 2.034–4.482, *P* < 0.001), location (OR, 95% CI: ICA, reference; ACA, 19.333, 7.868–54.677; MCA, 6.089, 2.424–17.262; PCOA, 10.411, 4.653–27.334; PC, 14.128, 5.598–40.472), size (OR: 1.289, 95% CI: 1.167–1.433, *P* < 0.001), neck width (OR: 0.775, 95% CI: 0.638–935, *P* = 0.008), and diabetes history (OR: 0.394, 95% CI: 0.159–0.877, *P* < 0.03).

**Table 1:** Univariate analysis of patient and aneurysm characteristics in the derivation cohort.

**Characteristics**	**Unruptured**	**Ruptured**	***P*-value**
	**(*n =* 414)**	**(*n =* 197)**	
**Patient characteristics**			
Age, years, mean (SD)	59.44 ± 9.46	58.19 ± 9.06	0.123
Gender (Male), *n* (%)	111 (26.8)	56 (28.4)	0.748
**Medical history**, ***n*** **(%)**			
Hypertension (yes)	271 (65.5)	127 (64.5)	0.881
Diabetes (yes)	39 (9.4)	8 (4.1)	0.031^*^
Hyperlipidemia (yes)	27 (6.5)	6 (3.0)	0.113
Atrial fibrillation (yes)	4 (1.0)	1 (0.5)	0.914
Coronary heart disease (yes)	36 (8.7)	19 (9.6)	0.817
SAH (yes)	8 (1.9)	2 (1.0)	0.621
Smoking (yes), *n* (%)	64 (15.5)	24 (12.2)	0.340
Drinking (yes), *n* (%)	22 (5.3)	11 (5.6)	1.000
**Morphological characteristics**			
Number of aneurysms			0.021^*^
2	242 (58.5)	138 (70.1)	
3	116 (28.0)	46 (23.4)	
4	36 (8.7)	8 (4.1)	
5	20 (4.8)	5 (2.5)	
Irregular shape (yes), *n* (%)	129 (31.2)	126 (64.0)	<0.001^*^
Neck width, mm, mean (SD)	3.33 (1.65)	3.58 (1.62)	0.078
Bifurcation location (yes), *n* (%)	37 (8.9)	19 (9.6)	0.894
Size, mm, mean (SD)	4.36 (3.12)	5.83 (2.95)	<0.001^*^
Location, *n* (%)			<0.001^*^
ACA/ACOA	45 (10.9)	45 (22.8)	
ICA	114 (27.5)	7 (3.6)	
MCA	69 (16.7)	23 (11.7)	
PCOA	150 (36.2)	93 (47.2)	
PC	36 (8.7)	29 (14.7)	

*SAH, subarachnoid hemorrhage; ACA, anterior cerebral artery; ICA, internal carotid artery; MCA, middle cerebral artery; PCOA, posterior communicating artery; PC, posterior circulation*.

* *Indicates a significant difference*.

**Table 2:** Results of multivariable logistic regression analysis.

**Predictors**	**Coefficient**	**Odds ratio (95% CI)**	***P* value**
Neck width	−0.255	0.775 (0.638–0.935)	0.008
Size	0.254	1.289 (1.167–1.433)	<0.001
Irregular shape	1.102	3.011 (2.034–4.482)	<0.001
**Location**			
ICA	Reference	Reference	Reference
ACA	2.962	19.333 (7.868–54.677)	<0.001
MCA	1.806	6.089 (2.424–17.262)	<0.001
PCOA	2.343	10.411 (4.653–27.334)	<0.001
PC	2.648	14.128 (5.598–40.472)	<0.001
Diabetes history	−0.932	0.394 (0.159–0.877)	0.030

*ACA, anterior cerebral artery; ICA, internal carotid artery; MCA, middle cerebral artery; PCOA, posterior communicating aneurysms; PC, posterior circulation*.

### Construction of Nomogram

**Figure 1:**
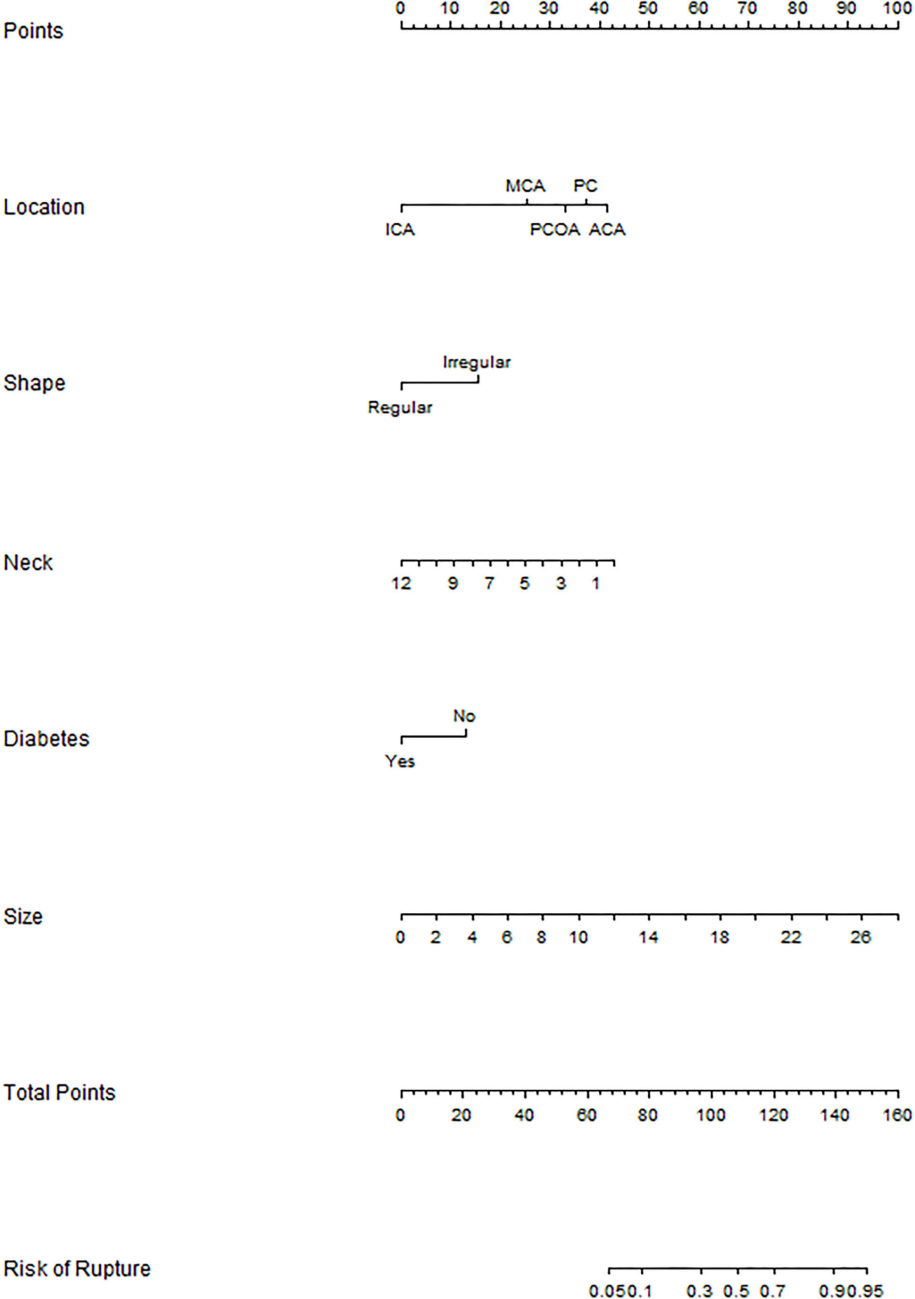
Nomogram for rupture risk assessment of multiple aneurysms. ICA, internal carotid artery; MCA, middle cerebral artery; PCOA, posterior communicating artery; PC, posterior circulation; ACA, anterior cerebral artery.

The binary logistic regression model was constructed by integrating the 6 independent risk factors into the numerical estimation of the probability of IA rupture: Log [*p*(*x*) /1—*p*(*x*)] = −0.255 ^*^ neck width +0.254 ^*^ size + 1.102 ^*^ irregular shape + 2.962 ^*^ ACA + 1.806 ^*^ MCA + 2.343 ^*^ PCOA + 2.648 ^*^ PC −0.932 ^*^ diabetes history-−3.726, where *p*(*x*) was the predicted probability of IA rupture. Then the model was converted into a graphic nomogram (Figure 1). For example, a patient with diabetes history (0 points) suffering from an aneurysm with a neck width of 2.6 mm (34 points), size of 5 mm (19 points), regular shape (0 points), and the location at ACA (40 points) would have a total score of 93, corresponding to an around 30% probability of rupture (Supplementary Figure S3). To facilitate the application of the nomogram model in clinical practice, we developed a dynamic nomogram which is a web-based calculator (Supplementary Figure S4) accessible for free on https://wfs666.shinyapps.io/onlinecalculator/.

### Performance of Nomogram

**Figure 2:**
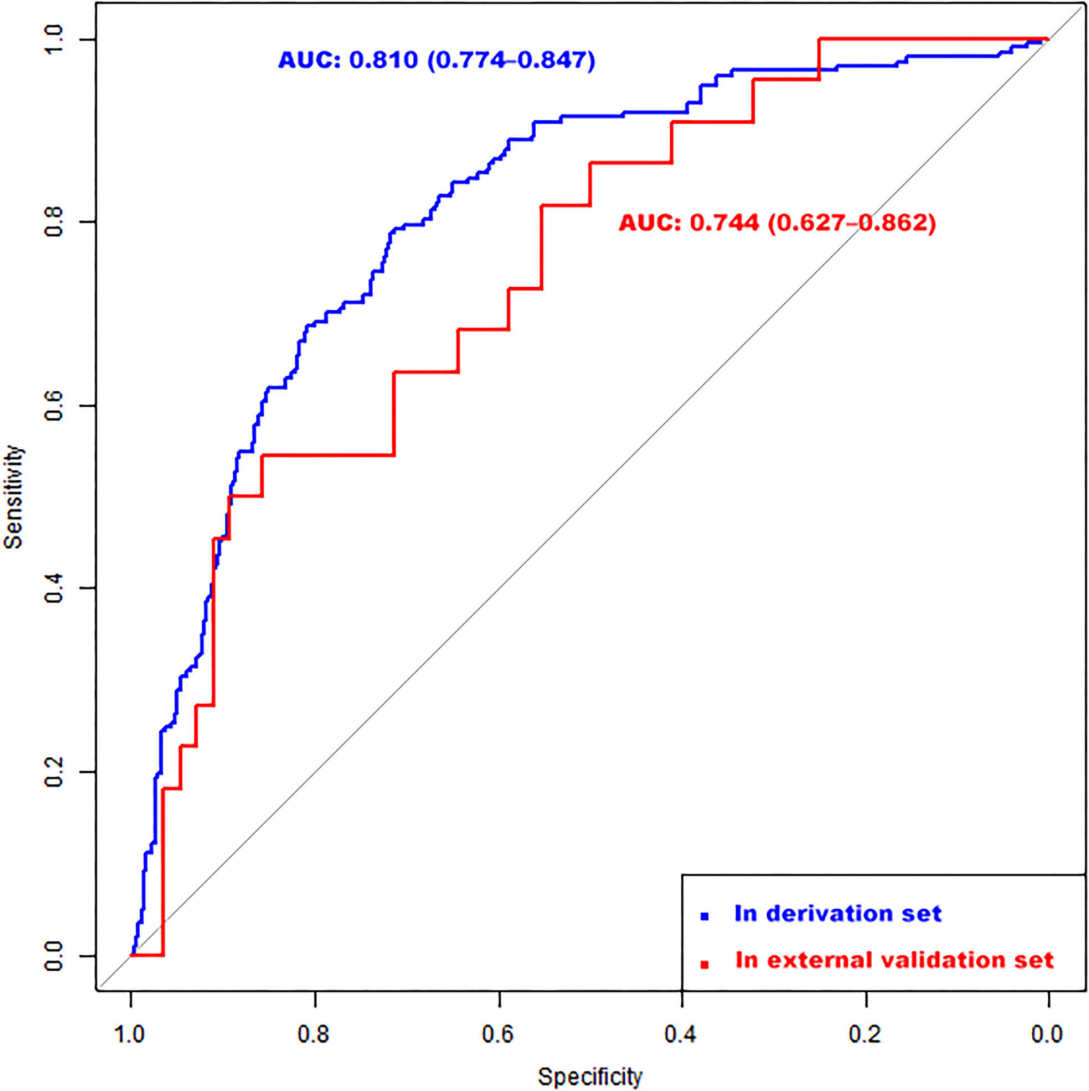
Receiver operating characteristic (ROC) curves of the nomogram for identifying high-risk IA among multiple aneurysms in the derivation (AUC = 0.81; 95% CI, 0.774–0.847) and external validation (AUC = 0.744; 95% CI, 0.627–0.862) set. CI, confidence interval.

**Figure 3:**
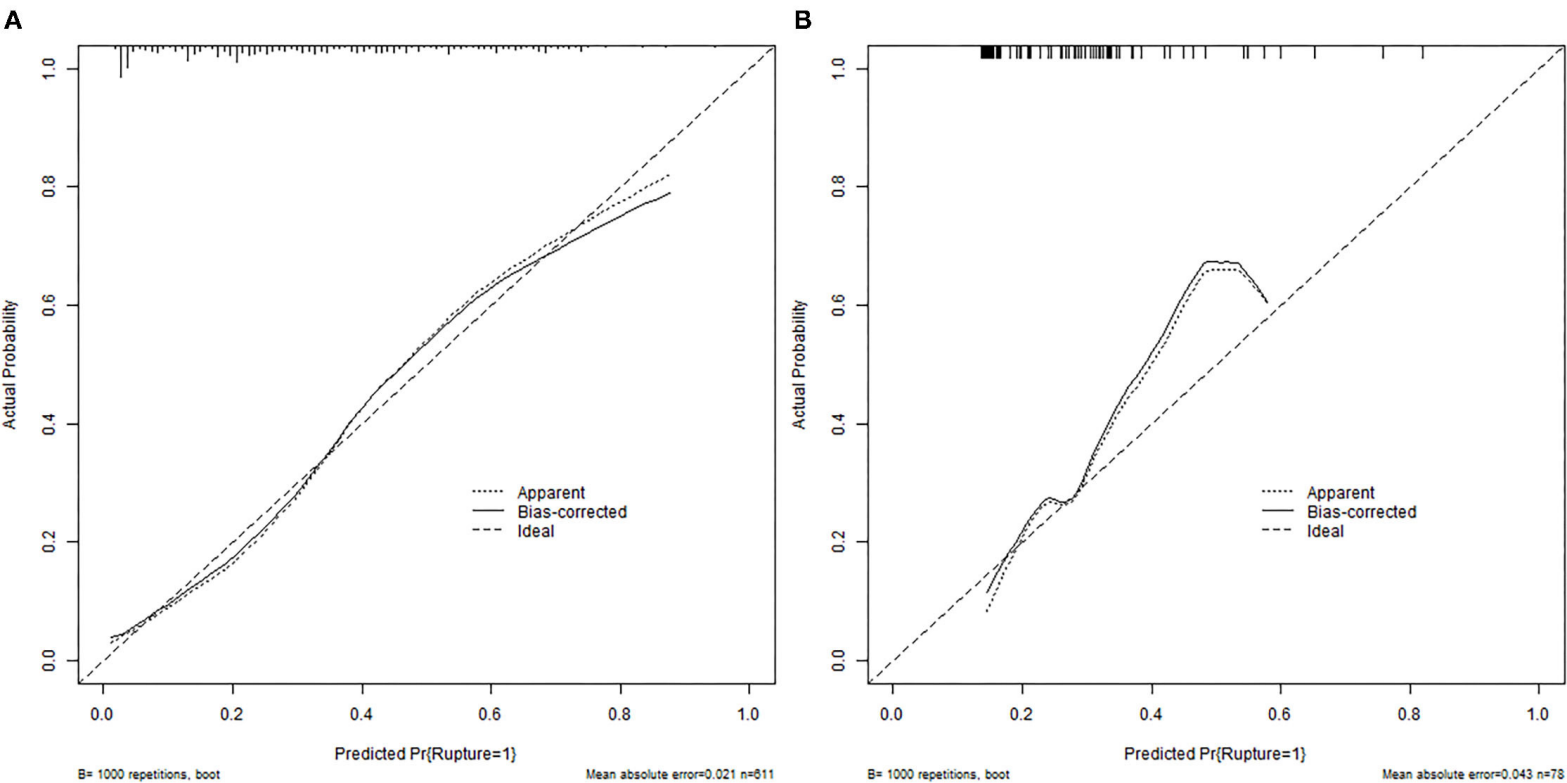
Calibration plots for the nomogram in training **(A)** and external validation **(B)** cohorts. The diagonal dashed line represents the ideal plot of the calibration plot. The dotted line represents the performance of the nomogram, while the solid line corrects for any bias in the nomogram.

**Figure 4:**
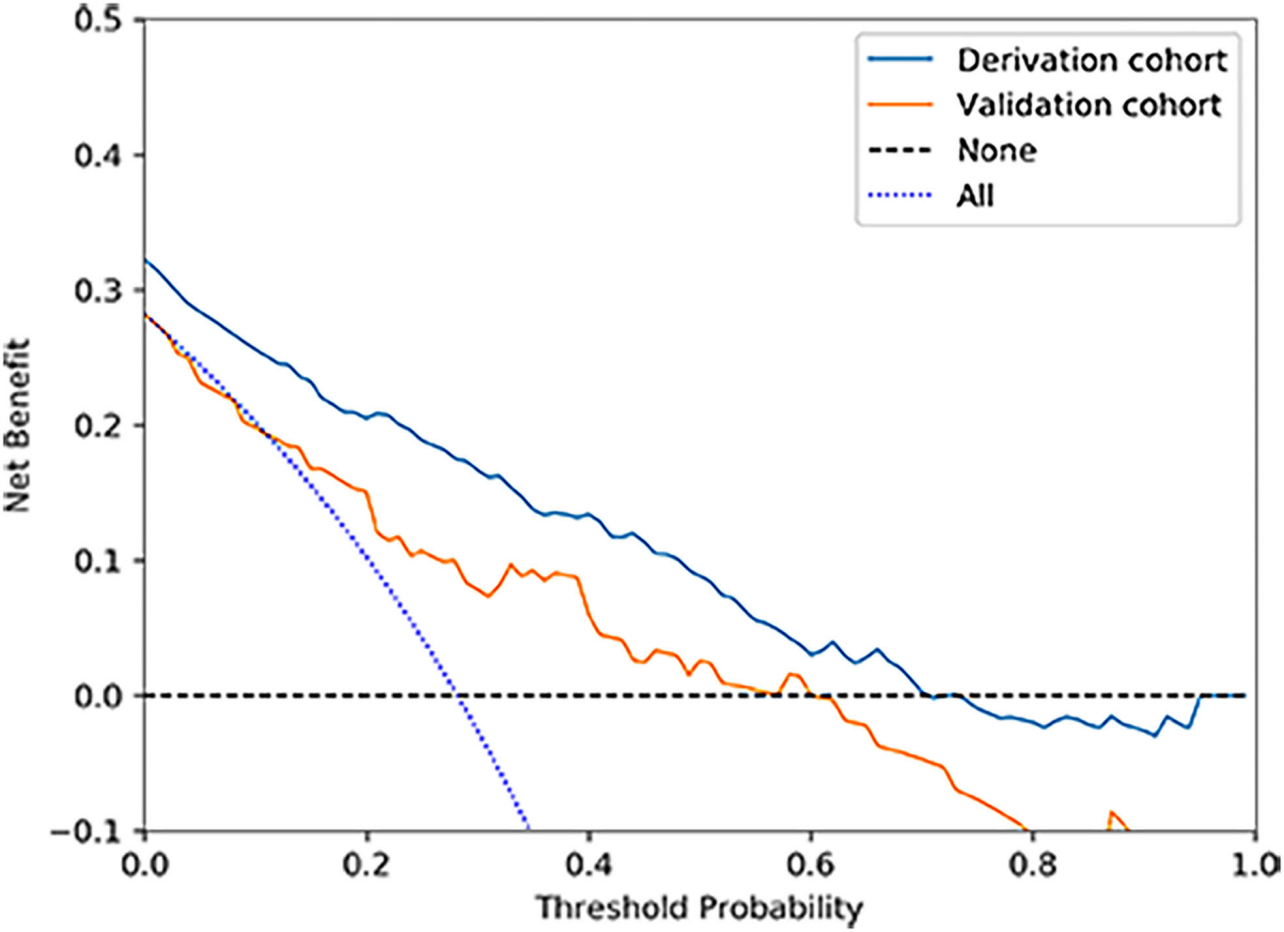
Decision curve analysis for the nomogram in derivation and validation cohorts. The nomogram to predict IA rupture would generate more NB than the “treat all” or “treat none” strategies and generate the maximum NB of about 0.18 and 0.26 for the derivation and validation cohort at the decision threshold of 0.1.

According to the ROC analysis, the nomogram showed good discriminative ability (Figure 2) both in the derivation (AUC = 0.81; 95% CI, 0.774–0.847) and external validation cohort (AUC = 0.744; 95% CI, 0.627–0.862). A calibration curve was plotted in the derivation (Figure 3A) and external validation (Figure 3B) set. The Hosmer–Lemeshow goodness-of-fit test demonstrated the nomogram had good calibration in the training (χ2 = 21.999, *P* = 0.005) and testing (χ2 = 7.6139, *P* = 0.472) sets, which indicated that the nomogram predicted probabilities of IA rupture was in good agreement with the actual probabilities. By decision curve analysis, the nomogram to discriminate IA rupture would generate more NB than the “treat all” or “treat none” strategies at the threshold probabilities ranging from 10 to 60% both in the derivation and external validation set (Figure 4). The nomogram generated the maximum NB of about 0.18 and 0.26 for the derivation and validation cohort at the decision threshold of 0.1.

To quantitatively compare with the widely used PHASE score, we calculated the PHASE score for the largest IA of patients with MIAs in our database. Among the 262 patients with MIA, 23% had a PHASES score of 0–6 points with an estimated 5-year rupture risk of <1%, 47% of all patients had a 5-year rupture risk of 1–1.9%, 21% of all patients had a 5-year rupture risk of 2–4.9% and 10% of all patients had a 5-year rupture risk of >5% (Supplementary Table S3). The PHASE score yielded a low AUC of 0.687 (0.619–0.755).

## Discussion

For the treatment strategy of unruptured IAs, there remains controversial, especially for unruptured MIAs. As a patient with MIAs is admitted for treatment, choosing the treatment all or other strategies without evaluation is clearly unreasonable. In this study, we constructed a dynamic nomogram model consisting of six variables easily available in routine clinical practice to evaluate the rupture risk of IAs among patients with MIA, which could be accessed for free on https://wfs666.shinyapps.io/onlinecalculator/. In general, combining our model and clinical experience could aid physicians to identify IAs with a high risk of rupture, reducing economic pressure for patients with a low risk of rupture.

Compared with the two present nomogram models, one superiority of our model was an external validation cohort from the distinct region and medical center. Indeed, as employed to new populations, most prediction models tended to perform worse than in their derivation and internal validation sets (16). Therefore, it is necessary to test the prediction model in an external validation set. In our study, the discrimination ability of our nomogram model was tested by a new validation cohort and achieved a good model performance, that is, an AUC of 0.81 and 0.744 in the derivation and external validation cohort, respectively. However, neither of the two present models were tested or validated by a new dataset: the GMB-MIAs nomogram model gained an AUC of 0.772 in the validation set (17), and another nomogram model presented a better performance in the validation set took advantage of morphology-based radiomics signature and traditional morphological data (11).

Another advantage of our nomogram model was a convenience to use in practice. The features contained by our model could be collected in routine clinical practice, which does not require physicians to take much time out of their busy schedules to acquire complex features. In addition, to further improve the convenience of our model in the increasingly intelligent clinical environment, we constructed an online calculator that allows clinicians and patients to use our nomogram model through the website directly and simply. Overall, our model could be a valuable and convenient tool to optimize clinical decisions and provide personalized treatment strategies and furnish additional information for assessing the rupture risk of MIAs based on the previous research.

Although aware of the deficiency of our dataset without follow-up, we consider the dataset is valuable for this study for several reasons. There is no doubt that prospective data with long-term follow-up are high-quality and ideal. Nonetheless, establishing a prospective cohort of patients with IAs is not reasonable enough to some extent. On the one hand, we cannot ignore these patients with high rupture risk IA(s), which was evaluated by present risk assessment methods (18). On the other hand, if only taking the remaining IAs with relatively low risk into observation and investigation, it will bring a great selection bias to the research. Moreover, there were several models had been published, which were constructed based on retrospective data without follow-up (11, 17). As a result, although data of ruptured IAs in our dataset emerged from IAs ruptured before admission, while not from long-term follow-up records, we still considered that it is beneficial to apply the dataset to develop our nomogram model.

A model including features that have been well investigated could be more reliable (8). In our nomogram model, all risk factors were confirmed by previous studies, including as follows: IAs size (19), location, shape (18, 20), diabetes history, and neck width. Similar to the former conclusion (21, 22), our study indicated that IAs located at PC, ACOA and POCA represented a dangerous signature of rupture. The effects in the natural course of IAs exerted by diabetes history have not been adequately considered. Several previous studies found the negative relationships between aneurysm rupture and diabetes history (23–25), which were consistent with our results. It may be interpreted as the effect of hypoglycemic agents in patients with diabetes (26), but further research is still required. Another interesting finding was that IAs with narrow neck shows a higher tendency to rupture. The ARETA study also considered the narrow neck of IAs as a risk factor linked to ruptured IAs (27) and indicated that further studies were acquired to reveal the underlying mechanisms.

Several limitations still existed in the present study. Firstly, this is a cross-sectional study based on retrospective datasets, which could only draw a preliminary conclusion on the rupture risk of multiple aneurysms, not a decisive conclusion. Secondly, our external validation cohort is relatively small scale, and further validation to the model is required in the future. Thirdly, for the sake of improving the convenience of our model, we ignored some factors associated with IAs rupture, such as hemodynamic parameters (28), patterns of wall enhancement (29). Besides, some important morphological parameters, especially size ratio and aspect ratio (10, 18), have not been included in our study. These may exert some influence on the performance of our model. Finally, this study is limited to the Chinese population, and country differences have not been taken into account (1, 30).

## Conclusion

A convenient dynamic nomogram model was established and achieved a good model performance in the cross-regional external validation cohort. It showed that the model had the potential to provide some help for clinical screening of more dangerous IAs in patients with MIAs after being evaluated by longitudinal data. Size, location, and neck width seemed to have the greatest impact on the outcome of the rupture.

## Data Availability Statement

The original contributions presented in the study are included in the article/Supplementary Material, further inquiries can be directed to the corresponding authors.

## Ethics Statement

The studies involving human participants were reviewed and approved by the Ethics Committee of Hunan Provincial People's Hospital ([2015]-10). The Ethics Committee waived the requirement of written informed consent for participation.

## Author Contributions

JZ, YL, and HF conceived and designed the study. WX, TC, and ZZ contributed equally to this work and conducted the literature review. TC performed data analysis. WX drafted the manuscript. XL, YS, LX, and DC collected the data. LG and ZZ polished this article. All authors have read and agreed to the published version of the manuscript.

## Funding

This study was funded by the Special Scientific Research Fund Project of Jiangsu Research Hospital Association—Lean Drug use—Stone Medicine [Grant Number JY202001] and National Natural Science Foundation of China [Grant Number 82173899].

## Conflict of Interest

The authors declare that the research was conducted in the absence of any commercial or financial relationships that could be construed as a potential conflict of interest.

## Publisher's Note

All claims expressed in this article are solely those of the authors and do not necessarily represent those of their affiliated organizations, or those of the publisher, the editors and the reviewers. Any product that may be evaluated in this article, or claim that may be made by its manufacturer, is not guaranteed or endorsed by the publisher.
