# Supplementary material for: Development and External Validation of a Dynamic Nomogram With Potential for Risk Assessment of Ruptured Multiple Intracranial Aneurysms
Source: Front Neurol. 2022 Feb 8;13:797709. doi: 10.3389/fneur.2022.797709 (PMC8861520; doi:10.3389/fneur.2022.797709)
Supplement: Supplementary file 1 [file Table_1.DOCX]

| **Table S1.** Comparison of characteristics between derivation and external validation cohort. | | | |
| --- | --- | --- | --- |
| **Characteristics** | **Derivation set (n=611)** | **Validation set (n=78)** | **P value** |
| Age, years, mean (SD) | 59.04 (9.35) | 61.79 (11.57) | 0.017* |
| Gender (Male), n (%) | 167 (27.3) | 17 (21.8) | 0.365 |
| Medical history, n (%) |  |  |  |
| Hypertension | 398 (65.1) | 46 (59.0) | 0.344 |
| Diabetes | 47 (7.7) | 12 (16.4) | 0.015* |
| Atrial fibrillation | 5 (0.8) | 2 (2.7) | 0.354 |
| Coronary heart disease | 55 (9.0) | 10 (12.8) | 0.378 |
| SAH | 10 (1.6) | 2 (2.6) | 0.897 |
| Smoking, n (%) | 88 (14.4) | 7 (9.0) | 0.256 |
| Drinking, n (%) | 33 (5.4) | 3 (3.8) | 0.756 |
| Number of aneurysms, n (%) |  |  | 0.030* |
| 2 | 380 (62.2) | 59 (75.6) |  |
| 3 — 4 | 206 (33.7) | 19 (24.4) |  |
| >4 | 25 (4.1) | 0 (0.0) |  |
| Bifurcation location, n (%) | 56 (9.2) | 7 (9.6) | 0.906 |
| Shape (Irregular), n (%) | 255 (41.7) | 18 (23.1) | 0.002 * |
| Neck width (Narrow), n (%) | 165 (27.0) | 19 (24.4) | 0.718 |
| Size, mm, mean (SD) | 4.84 (3.14) | 4.56 (3.68) | 0.491 |
| Location, n (%) |  |  | 0.055 |
| ICA | 121 (19.8) | 24 (30.8) |  |
| ACA | 90 (14.7) | 15 (19.2) |  |
| MCA | 92 (15.1) | 8 (10.3) |  |
| PCOA | 243 (39.8) | 21 (26.9) |  |
| PC | 65 (10.6) | 10 (12.8) |  |
| Ruptured status | 197 (32.2) | 22 (28.2) | 0.554 |
| SAH, subarachnoid hemorrhage; ACA, anterior cerebral artery; ICA, internal carotid artery; MCA, middle cerebral artery; PCOA, posterior communicating artery; PC, posterior circulation. | | | |


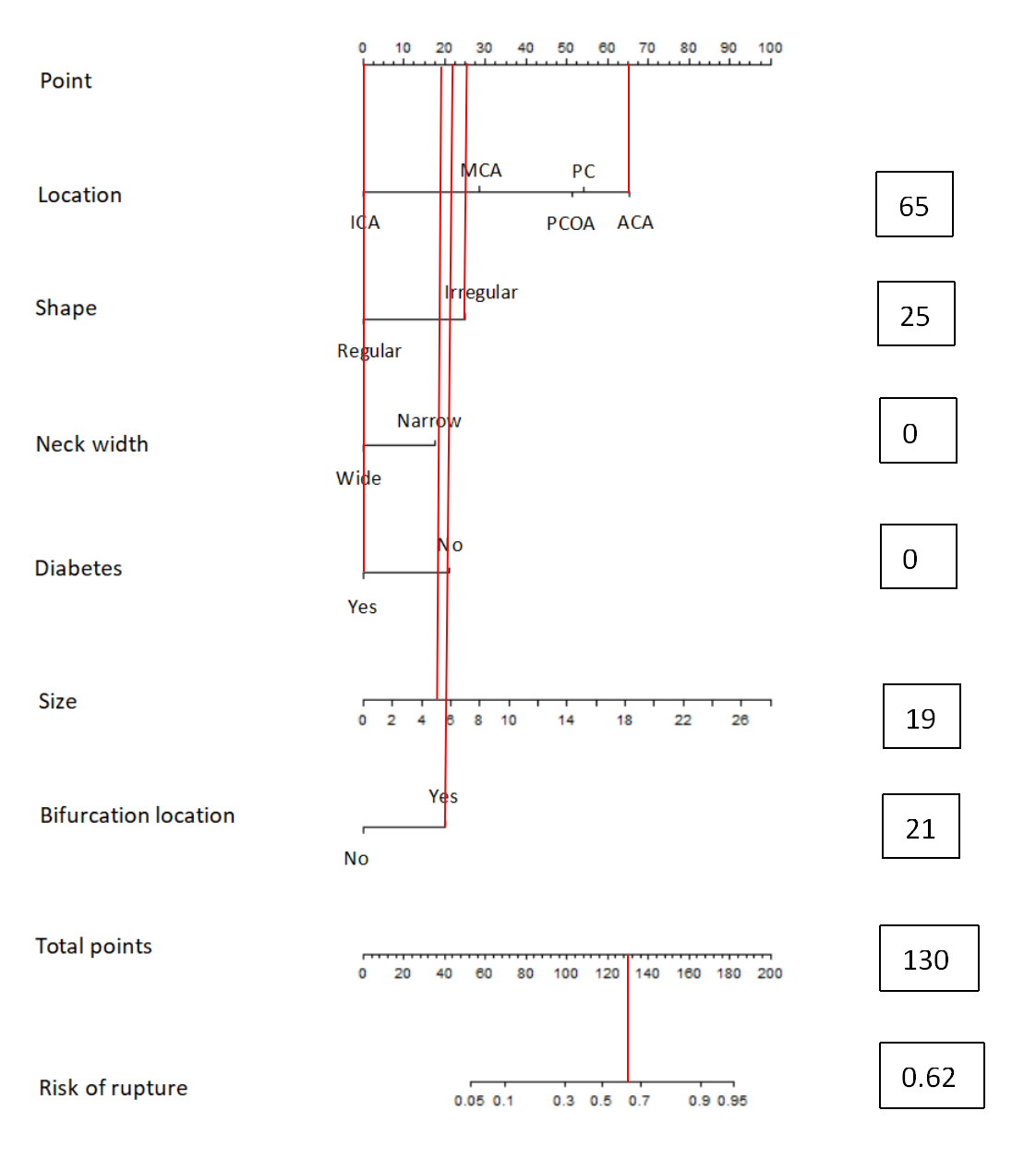


**Figure S1.** An example of using the nomogram to calculate the probability of aneurysm rupture in patients with multiple intracranial aneurysms.


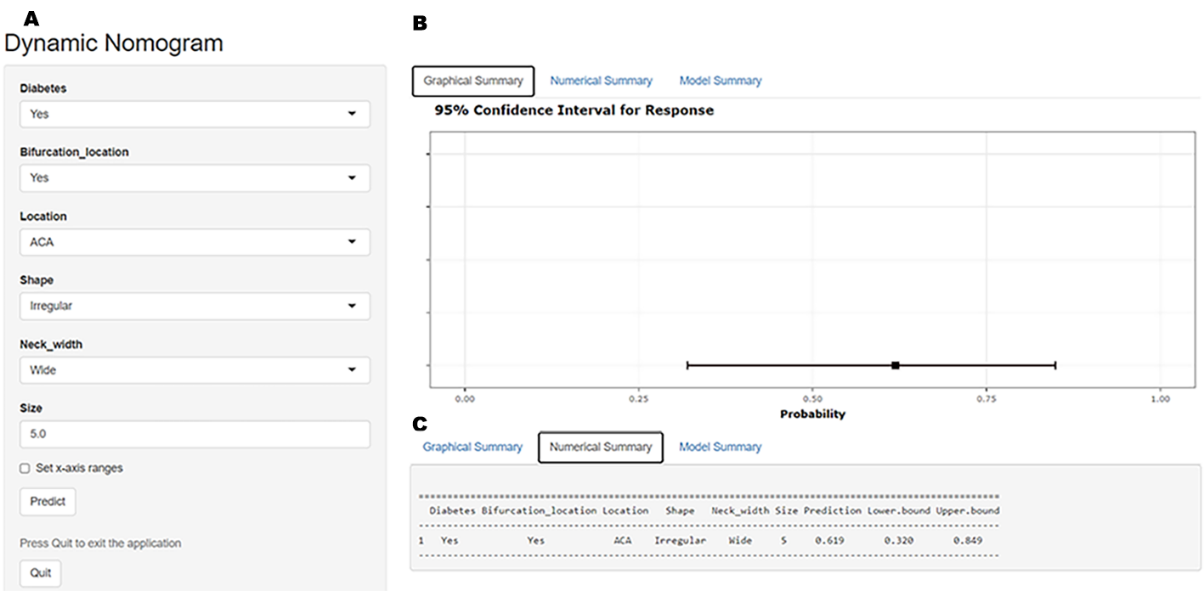


**Figure S2.** Web-based dynamic nomogram for assessing aneurysm rupture risk among multiple aneurysms. Enter history of DM, bifurcation location, location, shape, neck width and size of patients in part A of <https://bb66351ctt.shinyapps.io/MIArupture/>, and get the prediction result in part B and C. Graphical summary: It shows the rupture probability of the aneurysm and 95% confidence intervals. Numerical summary: It represents the calculated value of the rupture probability and 95% confidence intervals.
